# Supplementary material for: Serum Antibodies Against the E5 Oncoprotein from Human Papillomavirus Type 16 Are Inversely Associated with the Infection and the Degree of Cervical Lesions
Source: Biomedicines. 2024 Nov 26;12(12):2699. doi: 10.3390/biomedicines12122699 (PMC11673199; doi:10.3390/biomedicines12122699)
Supplement: Supplementary file 1 [file biomedicines-12-02699-s001.zip › biomedicines-3273245-supplementary.pdf]

Supplementary Figure S1

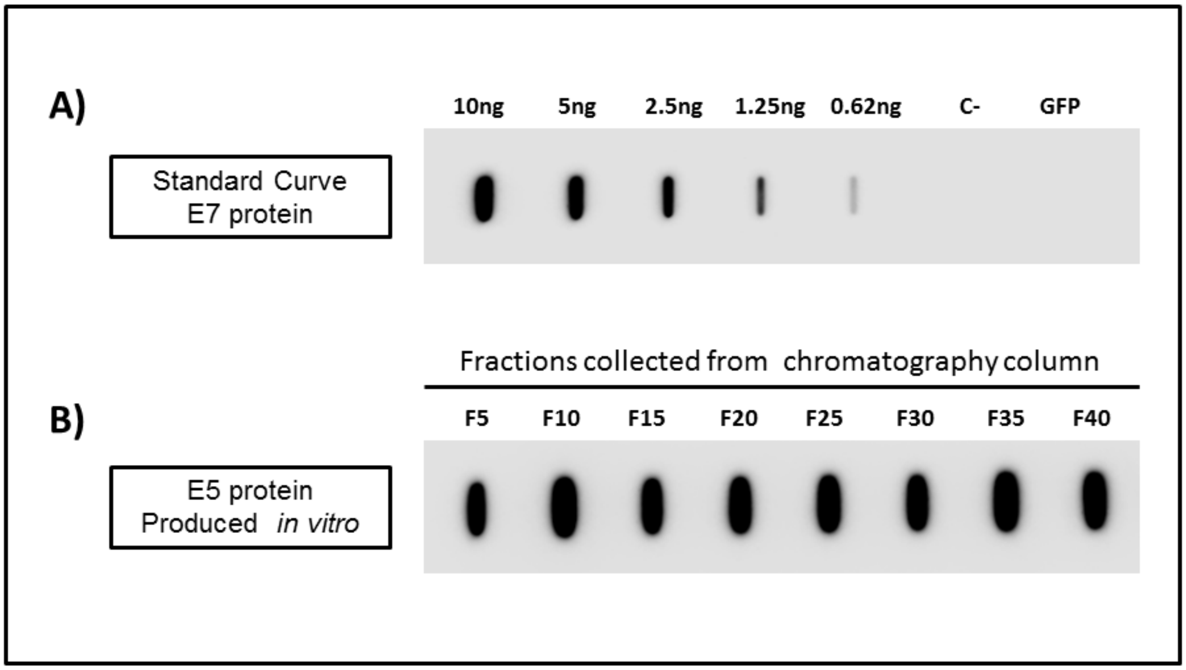

Supplementary Figure S1. **Calibration curve to calculate E5 protein concentration.** A) A stock of HPV16 E7 protein of known concentration was used to prepare a standard protein concentration curve (from 10 ng to 0.625 ng). Protein was detected with an anti-E7 mouse monoclonal antibody (dil. 1:1000) (ED17, Santa Cruz). The elution buffer (C-) and green fluorescent protein (GFP) were used as negative controls. B) Ten microliters of collected fractions containing E5 protein (F5 to F40), produced *in vitro*, were detected with the in-house anti-His mouse monoclonal antibody 2R2A6 (dil. 1: 1000). The blots were developed by chemiluminescence and analyzed in the Odyssey Fc® system. The concentration of E5 in the fractions was calculated from the E7 standard curve.

Supplementary Figure S2

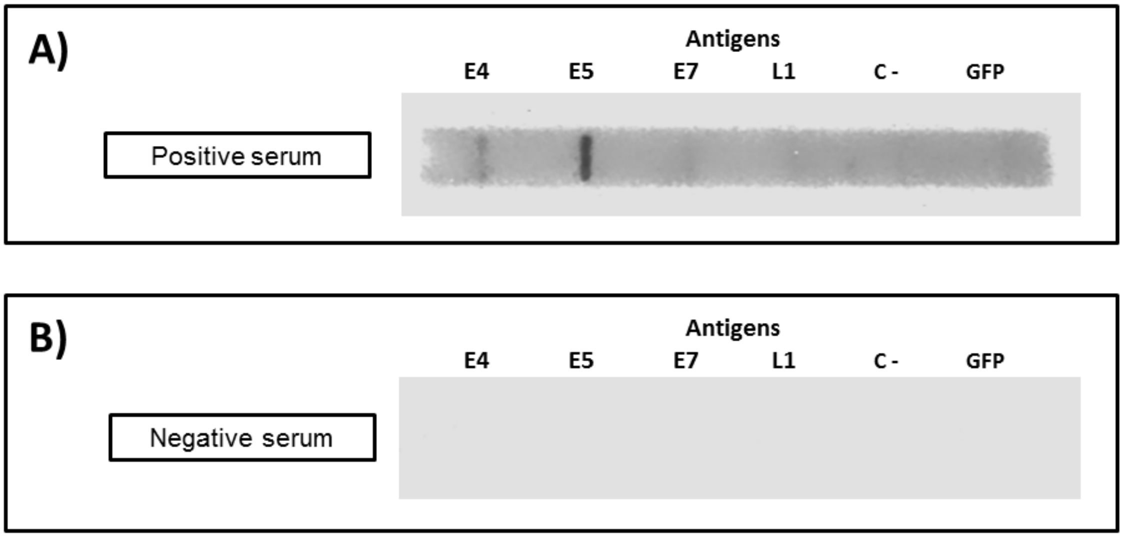

Supplementary Figure S2. Seropositivity to E5 protein in sera from women in the study population was analyzed by slot blot. Ten ng of E5 protein and other HPV antigens were immobilized in PROTRAN membranes using the Hybri-Slot system. The strips containing the antigens, the negative controls buffer (C-), and the green fluorescence protein (GFP) were incubated with different serum from the study population (dil. 1:2,500). The slot blots were developed with the amplifying biotin-streptavidin system, treated for chemiluminescence, and analyzed with the Odyssey Fc® system. The strips show examples of one positive (A) and one negative (B) serum for the presence of anti-E5 antibodies. Both sera were taken from the No Lesion group of the population of women studied.

Supplementary Figure S3

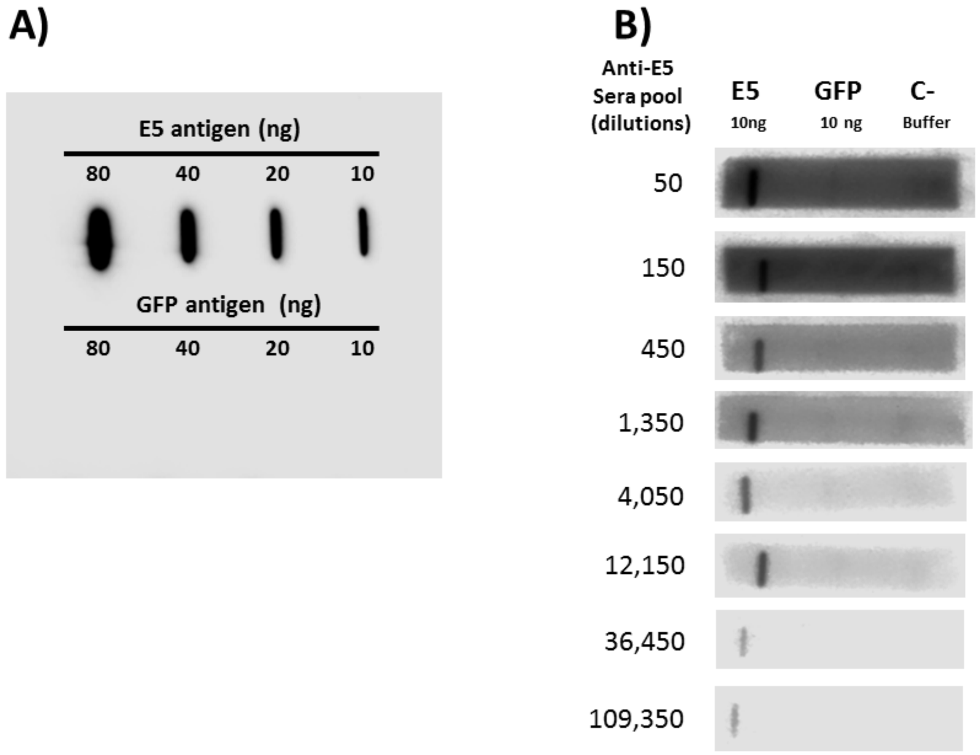

Supplementary Figure S3. Specificity and titration of positive control sera for anti-E5 antibodies. A) The positive control anti-E5 serum was tested by slot blot with different concentrations of HPV16 E5 protein produced *in vitro* and against GFP. The serum was tested at a dilution of 1:2,000 and the secondary biotinylated antibody at a dilution of 1:10,000 and developed by chemiluminescence and analyzed with the Odyssey Fc® system. B) Slot blot titration curve for anti-E5 antibodies was generated by testing serial dilutions of the pool of anti-E5 antibody positive sera against 10 ng of HPV16 E5 antigen and GFP (negative control). Strips were developed with the biotin-streptavidin secondary antibody system and chemiluminescence. Blots were analyzed with the Odyssey Fc® system.
